# Gestational Psittacosis With Secondary Hemophagocytic Syndrome: A Case Report and Literature Review

**DOI:** 10.3389/fmed.2021.755669

**Published:** 2021-11-18

**Authors:** Li Sun, Pulin Li, Bo Pang, Peipei Wu, Ran Wang

**Affiliations:** Department of Respiratory and Critical Care Medicine, The First Affiliated Hospital of Anhui Medical University, Hefei, China

**Keywords:** gestational psittacosis, hemophagocytic syndrome, metagenomic next-generation sequencing, gestational psittacosis with secondary hemophagocytic syndrome, long-term high fever

## Abstract

Gestational psittacosis and hemophagocytic syndrome (HPS) are rare clinical diseases. In this article, a case of gestational psittacosis concomitant with secondary HPS was reported. An analysis was performed on the clinical characteristics, signs, laboratory findings, progression, diagnosis, and treatment of a patient with gestational psittacosis concomitant with secondary HPS. Besides, the literature with respect to this disease was reviewed. This patient was definitively diagnosed through metagenomic next-generation sequencing techniques, bone marrow puncture and smear examination, and the determination of sCD25 level and natural killer (NK) cell activity. Anti-infectives such as doxycycline and etoposide combined with hormone chemotherapy achieved significant improvement in cough and expectoration, a return to normal temperature, and a significant improvement in oxygenation index. In addition, chest computed tomography revealed obvious absorption of lung lesions and a return of NK cell activity and sCD25 levels to normal ranges. *Chlamydia psittaci* pneumonia requires a clear determination of etiology, while HPS requires bone marrow puncture and smear examination, together with the determination of sCD25 level and NK cell activity in the blood. The findings of this study suggest that metagenomic next-generation sequencing is an effective instrument in clearly identifying pathogens that cause lung infection. Clinicians should consider atypical pathogens of lung infection in patients with poor response to empirical anti-infectives, and strive to design an effective treatment strategy as per an accurate diagnosis based on the etiology. As for patients suffering from long-term high fever and poor temperature control after broad-spectrum antibiotic treatment, non-infectious fever should be taken into account. A rapid and clear diagnosis would significantly improve patient prognosis.

## Introduction

Intimate contact with birds is usually associated with a risk of suffering from anthropozoonosis caused by *Chlamydia psittaci*. C. *psittaci* pneumonia is a rare clinical disease, with an incubation period of 1–4 weeks. The clinical manifestations of this disease include influenza and high fever with a temperature more than 40°C, accompanied by headache, myalgia, joint pain, and other symptoms, including cough, expectoration, and bloody sputum occurring ~1 week after the infection. *C. psittaci* infection can involve multiple systems, among which the pulmonary organs are the main targets. Therefore, respiratory failure may occur in severe cases (1). Hemophagocytic syndrome (HPS), also known as hemophagocytic lymphohistiocytosis (HLH), is a type of excessive inflammatory response syndrome caused by primary or secondary immune abnormalities. Most cases would develop disease secondary to infection, tumor(s), and/or autoimmune diseases (2). Infection can be caused by bacteria, parasites, and viral infections, including Epstein–Barr virus and cytomegalovirus. HPS is characterized by acute onset, rapid progress, difficult to treat, and high mortality. *C. psittaci*, which belongs to the genus *Chlamydia*, together with *Chlamydia pneumoniae* and *Chlamydia trachomatis*, can survive for several months at room temperature, and is an anthropozoonosis (3). Gestational psittacosis with secondary HPS is even more rare. To our knowledge, there is currently no report on gestational psittacosis concomitant with secondary HPS. Therefore, a case of gestational psittacosis with secondary HPSs is expounded in this article.

## Case Report

**Figure 1:**
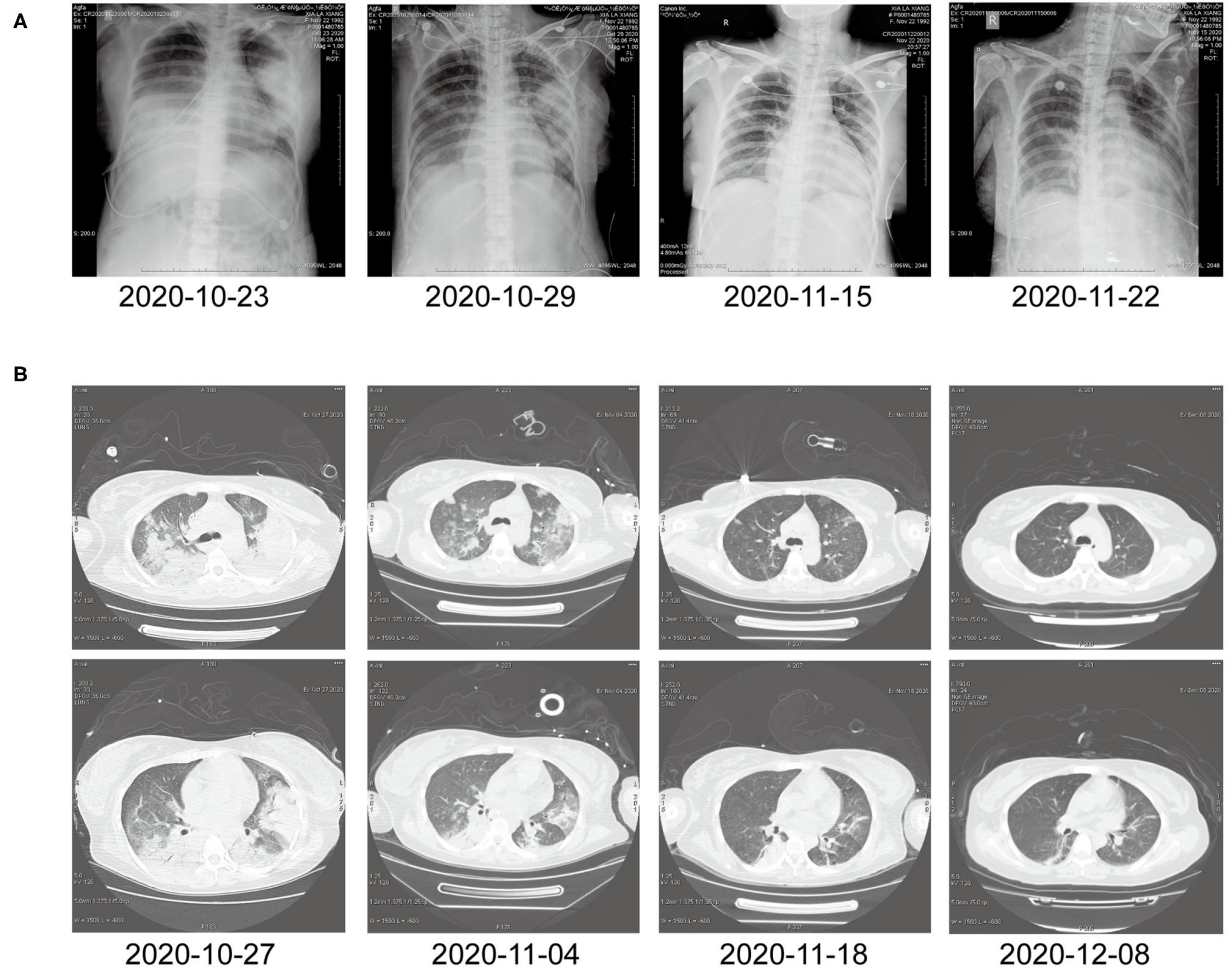
**(A)** Chest X-ray before and after treatment. **(B)** Chest computed tomography at different time points after treatment.

**Figure 2:**
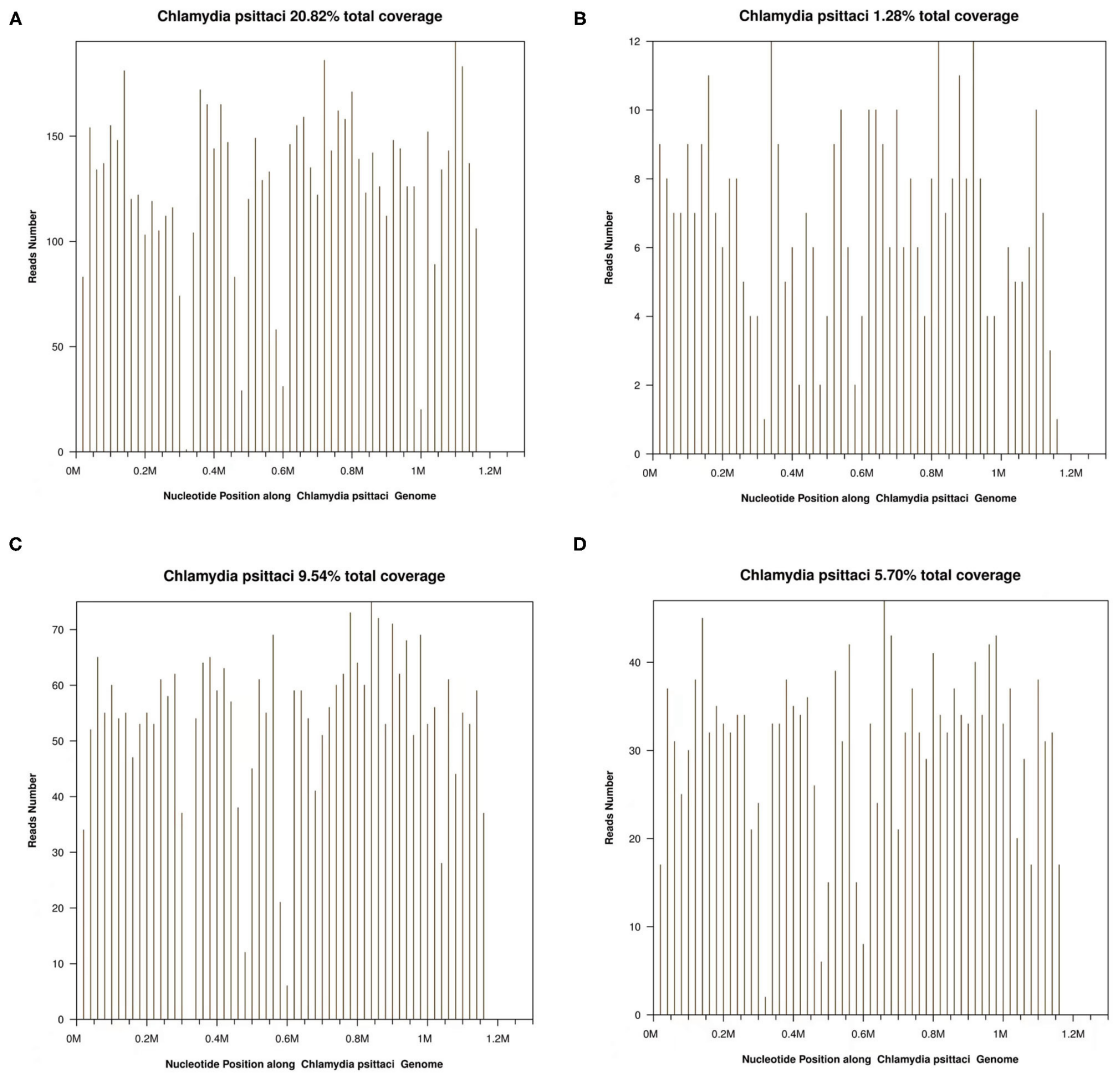
**(A)** *Chlamydia psittaci* detection in bronchoalveolar lavage fluid (BALF) using metagenomic next-generation sequencing (mNGS) on October 25, 2020. **(B)** *C. psittaci* in blood detected by mNGS on October 25, 2020. **(C)** *C. psittaci* detected in BALF using mNGS on November 4, 2020. **(D)** *C. psittaci* in blood detected by mNGS on November 4, 2020.

**Figure 3:**
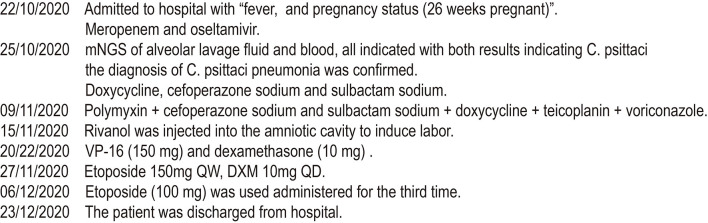
Timetable used to show the progress of the disease and the patient's treatment.

In this article, a case involving a 27-year-old woman with gravida 2, para 1, and gestational weeks of 26 was reported. This patient suffered from a fever shortly after she became soaked in the rain 6 days previously, with the highest temperature of 40°C. Then, she had cough 3 days previously, with white phlegm and occasional bloody sputum, combined with chest tightness and asthma. These symptoms were not improved after the administration of mezlocillin sodium and sulbactam sodium as anti-infectives in the outpatient department. Subsequently, she visited the emergency department of the First Affiliated Hospital of Anhui Medical University (Anhui, China) on October 22, 2020, where she was admitted to the hospital with “fever and pregnancy status (26 weeks pregnant).” This patient developed intermittent diarrhea in the previous 3 days, with the clinical manifestations of yellow watery stool and dark urine. The patient was previously in good condition and had no other diseases. Through auscultation, breathing sounds in both lungs were thick, with obvious moist rales. On the admission day, the temperature of the patient was 38.5°C, with a heart rate of 150 beats/min, a blood pressure of 105/69 mmHg, and a respiratory rate of 35 breaths/min. The findings of laboratory examinations were as follows: hemoglobin, 88 g/L; C-reactive protein (CRP), 145.46 mg/L; white blood cell count (WBC), 14.73 × 10^9^/L; platelet count, 189 × 10^9^/L; procalcitonin (PCT), 19.36 ng/mL; D-dimer, 12.85 μg/mL; glutamic-oxaloacetic transaminase, 125 U/L; and creatinine, 39.3 μmol/L. Chest X-ray (Figure 1A) revealed bilateral lung inflammation, lung abscess in the superior lobe of the left lung, and right pleural effusion. The preliminary differential diagnosis included severe pneumonia, acute respiratory distress syndrome, septic shock, and pregnancy. Although meropenem and oseltamivir were administered to resist infection, the patient's dyspnea symptoms persisted, and her high-flow oxygenation index was < 100 mmHg. After endotracheal intubation, the patient was transferred to the intensive care unit (ICU). On October 23, 2020, the patient was examined through fiberoptic bronchoscopy, sputum and alveolar lavage fluid in the left and right bronchi were aspirated, and meropenem, vancomycin, azithromycin, and oseltamivir were administered with mechanical-assisted ventilation. On October 25, 2020, the patient received metagenomic next-generation sequencing (mNGS) (Figures 2A,B) of alveolar lavage fluid and blood, with both results indicating *C. psittaci*. Therefore, the diagnosis of *C. psittaci* pneumonia was confirmed, and the antibiotic regimen was adjusted to include doxycycline, cefoperazone sodium and sulbactam sodium, and moxifloxacin. Symptomatic treatment, including mechanical ventilation, anti-shock, protection of important organs, nutritional support, sedation, and analgesia, was actively administered. On October 29, 2020, bronchoscopy was performed again, with the results of metagenomic next-generation sequencing (mNGS) of alveolar lavage fluid and blood continuing to show *C. psittaci*. However, the level of *C. psittaci* (Figures 2C,D) in the alveolar lavage fluid decreased compared to the earlier analysis, although the level of *C. psittaci* in the blood increased. On October 31, 2020, the oxygenation index of the patient reached 280 mmHg, which was significantly improved, the temperature of the patient was 40°C, with a heart rate of 105 beats/min and a blood pressure of 120/60 mmHg. The findings of laboratory examinations were as follows: hemoglobin, 93 g/L; CRP, 80.19 mg/L;WBC, 14.36 × 10^9^/L; platelet count, 140 × 10^9^/L; PCT, 4.87 ng/mL; D-dimer, 8.35 μg/mL; glutamic-oxaloacetic transaminase, 54 U/L; and creatinine, 26.7 μmol/L. Chest X-ray revealed improved bilateral lung inflammation and lung lesions compared with the condition at admission. However, the peak temperature of this patient was higher than before. As for those patients suffering from long-term high fever, if the temperature is not effectively controlled after extensive coverage with broad-spectrum and powerful antibiotics, non-infectious fever should be taken into account at the same time. In other cases, non-infectious fever is common in the rheumatic blood system and tumor diseases. Bone marrow puncture and smear examination were performed on November 2, 2020, and the tumor index of the patient was elevated, which was considered to be related to pregnancy and hypoproteinemia. Sputum and blood cultures showed *Acinetobacter baumannii*, and sputum and urine cultures showed *Candida albicans* and *Candida tropicalis*. Therefore, the antibiotic regimen was adjusted to include polymyxin + cefoperazone sodium and sulbactam sodium + doxycycline + teicoplanin + voriconazole on November 9, 2020. On November 15, 2020, ultrasound indicated that the umbilical cord was wound around the neck of the fetus for 2 weeks, the volume of amniotic fluid was decreased, and fetal kidney parenchyma echo was enhanced. After communicating with the family members of the patient and obstetrics physicians, rivanol was injected into the amniotic cavity to induce labor. On November 19, 2020, the patient became conscious, and her breathing and circulation were stable; however, she developed a cough reaction, and tried to break away from the ventilator and remove tracheal intubation. Pulmonary imaging revealed mitigated fever (temperature > 38.5°C), reduced red blood cell (RBC) count (1.99 × 10^12^/L) and platelet count (111 × 10^9^/L), hypertriglyceridemia (triglycerides, 6.21 mmol/L), and hyperferritinemia (ferritin, 2,284 μg/L). Phagocytic cells were apparent on bone marrow smear and HPS could not be excluded. Besides, natural killer (NK) cell activity and soluble CD25 levels were examined, and the CD107a excitation test was improved. These results indicated that the level of sCD25 increased, and NK cell activity decreased. Therefore, the diagnosis of HPS caused by infection was confirmed. The HLH-2004 regimen, recommended by the International Histocyte Association, was applied in the treatment of HPS. Etoposide combined with hormone therapy can significantly mitigate—if not eliminate—symptoms, and immunosuppressants, such as cyclosporine A (CSA) and anti-thymocyte globulin (ATG), could be added according to the condition (4). On November 20, 2020, VP-16 (150 mg) and dexamethasone (10 mg) were administered to eliminate symptoms. On November 22, 2020, the temperature of the patient returned to 36.8°C.On November 27, 2020, the temperature of the patient was basically maintained within the normal range. The finding of laboratory examinations were as follows: hemoglobin, 92 g/L; CRP, 7.48 mg/L;WBC, 11.43 × 10^9^/L; platelet count, 103 × 10^9^/L; PCT, 0.11 ng/mL; D-dimer, 0.93 μg/mL; glutamic-oxaloacetic transaminase, 62 U/L; creatinine, 13.3 μmol/L; RBC count, 2.83 × 10^12^/L; hypertriglyceridemia (triglycerides, 5.68 mmol/L); and hyperferritinemia (ferritin, 2,212 μg/L). The indices of ferritin and triglycerides for the patient did not decrease significantly compared with the previous time, and HPS had not been completely controlled. On November 27, 2020, etoposide (100 mg) was administered on the basis of dexamethasone to control the primary disease. The findings of laboratory examinations on December 3, 2020, were as follows: WBC, 6.55 × 10^9^/L; platelet count, 155 × 10^9^/L; triglycerides, 2.34 mmol/L; ferritin, 1,807 μg/L; and glutamic-oxaloacetic transaminase, 14 U/L. Etoposide (100 mg) was administered for the third time on December 6, 2020. On December 10, 2020, NK cell activity and sCD25 levels were re-examined, with the results showing that NK cell activity did not decrease and the level of sCD25 returned to the normal range. Therefore, it was not necessary to continue the treatment with etoposide, and dexamethasone was gradually decreased. On December 23, 2020, the fever, cough, and expectoration were obviously mitigated and breath sounds in both lungs became clear. Chest CT (Figure 1B) revealed that the lung lesions were absorbed, and the level of sCD25 and NK cell activity suggested that the condition of the patient was basically stable; therefore, the patient was discharged from the hospital. The telephone follow-up 1 week later revealed that the patient had no complaints of discomfort, fever, cough, or expectoration, and she was advised to conduct outpatient reviews regularly (Figure 3).

## Discussion

The pregnant woman reported that she had a history of contact with poultry a week before the onset of illness. Also, it was found from the results of mNGS sent by bronchoalveolar lavage fluid (BALF) that she was infected with *C. psittaci*. After active anti-infective treatment, the lung lesions of the patient were significantly absorbed. However, she still had a high fever, reduced RBC and platelet count, hypertriglyceridemia, hyperferritinemia, apparent phagocytic cells on bone marrow smear, increased level of sCD25, and decreased activity of NK cells, and hence the diagnosis of gestational psittacosis with secondary HPS could be confirmed.

*Chlamydia psittaci* infections occur mainly in the autumn, and almost all relevant cases are associated with a fever. There are also other symptoms, including chills, myalgia, headache, diarrhea, dyspnea, and hemoptysis. *C. psittaci* pneumonia accounts for ~1% of community-acquired pneumonias (5). Sixty percent of patients with *C. psittaci* pneumonia exhibit dysfunction of the liver (6), as did the patient described in this article. The findings of laboratory examinations on October 25, 2020, were as follows: alanine aminotransferase (ALT), 88 U/L; aspartate aminotransferase (AST), 433 U/L; direct bilirubin, 22.6 μmol/L; and indirect bilirubin 38.3 μmol/L. After treatment, laboratory examinations were performed on November 2, 2020, with the results were as follows: ALT, 14 U/L; AST, 30 U/L; direct bilirubin 10.7 μmol/L; and indirect bilirubin 14.2 μmol/L. Ito et al. drew a comparison between mycoplasma pneumonia, *C. pneumonia*, and *C. psittaci* pneumonia, and they found that *C. psittaci* infection was dominated by frosted glass shadow bilateral lung lesions which were more common, and ~8% of patients exhibited pleural effusion (7). In this case, the patient exhibited bilateral lung disease with right pleural effusion. It has been demonstrated in some studies that the level of D-dimer in patients with *C. psittaci* pneumonia concomitant with single or multiple lung lobe involvement is significantly higher than that in those with simple lung segment lesions, and the level of D-dimer can be regarded as a prognostic factor, which can be employed to reveal the changes in the condition of patients (8). There were also significant changes in the level of D-dimer which was evident in the present case, with the value exceeding 20 μg/mL at the initial stage of the disease. After the treatment with doxycycline, moxifloxacin, and other antibacterial drugs, the lung lesions in the patient decreased, and the level of D-dimer decreased to 4.17 μg/mL on November 2, 2020. Severe *C. psittaci* pneumonia is similar to *Legionella pneumonia* with regard to extrapulmonary manifestations, biological characteristics, and prognosis. The difference is that *C. psittaci* pneumonia is associated with a history of exposure to sick birds and a prolonged duration of symptoms before admission (9). In this case, the patient (a 27-year-old woman) had a history of contact with poultry 1 week before the onset of the disease.

At present, the diagnosis of *C. psittaci* pneumonia is mainly based on the contact history with sick birds, and pathogens in bronchial secretions. Currently, the detection methods include pathogen isolation and identification, immunofluorescence, complement fixation test, ELISA, routine PCR, and fluorescence PCR. The micro-immunofluorescence method is a common method to detect *C. psittaci*, and requires the sample collection twice at the acute and recovery stages. It is a retrospective diagnosis, which can be employed to determine the disease condition and evaluate the exposure situation. However, it is difficult to implement the culture technology for *C. psittaci* and excessive handling of samples may lead to contamination. Therefore, the high-level biosafety is required, which further limits clinical application. The detection of *C. psittaci* nucleic acid in respiratory secretions, blood, and tissues of patients with suspected acute-stage infection is a rapid and reliable diagnostic method with high sensitivity and specificity (10, 11). Moreover, nucleic acid detection can also provide strain genotyping and avoid cross-reactions with serological diagnosis (12). The recently developed mNGS is a novel technology to detect/identify microorganisms, and features high pathogen-positive detection rate, rapidity, wide pathogen coverage, and high sensitivity. It has been widely applied in the field of metagenomic detection of unknown pathogens, including hereditary diseases, tumors, infectious diseases, human leukocyte antigen analysis, and non-invasive prenatal screening (13). At present, mNGS is a favorable method for detecting unknown pathogens in pulmonary infections. The patient in this report suffered from severe infection, but no abnormality was found through routine detection of sputum etiology, and the empirical anti-infection treatment took no effect, in combination with the contact history of the patient with poultry 1 week before the onset of this disease. Bronchoscopy and alveolar lavage were utilized to collect BALF, and blood was collected for mNGS. The results identified *C. psittaci* as the pathogen, which provided clear direction for subsequent treatment. Currently, there are a few reports on the detection of *C. psittaci* through second-generation sequencing methods (14, 15). However, it has been reported in some studies that tetracyclines are the first-choice treatment for patients with *C. psittaci* infection (16). Azithromycin has also exhibited favorable activity against *C. psittaci*, while fluoroquinolones have a weak inhibitory effect, but can be used as an alternative (15). In this case, after 2 days of treatment with doxycycline, the temperature and oxygenation levels of the patient exhibited no significant improvement. Considering the poor anti-infective effect on the patient and the favorable therapeutic effect of moxifloxacin on *C. psittaci* pneumonia (16), antibiotic treatment was replaced with doxycycline, cefoperazone sodium, and sulbactam sodium combined with moxifloxacin. After 1 week of treatment, the condition of the patient was significantly improved, with specific manifestations of decreased inflammation index and absorbed lung lesions as per the chest CT results.

Hemophagocytic syndrome is a syndrome involving multiple systems, and it can be caused by the abnormal proliferation of activated macrophages and histiocytes (17). Its common clinical manifestations include high fever, enlarged liver, splenic lymph nodes, pancytopenia, low fibrinogen level, hypertriglyceridemia, abnormal liver enzyme levels, poor prognosis, and a high mortality rate (18). In this case, the patient also had a fever, with the temperature reaching 40°C, RBC count decreasing to 1.67 × 10^12^/L, platelet count decreasing to 75 × 10^9^/L, triglyceride levels reaching 4.38 mmol/L, and abnormal liver enzyme levels.

Hemophagocytic syndrome is associated with many diseases, and its etiology is particularly important in the diagnosis due to the fact that these potentially relevant diseases would exert significant impacts on the treatment and prognosis of individuals with HPS (19). HPS occurs rarely in pregnant women, and there are only a few studies about pregnancy-related HPS. In these published cases, it is unclear whether the cause of HPS was related to pregnancy itself or viral infection, or both (20). HPS in the patient was considered to be secondary to severe *C. psittaci* pneumonia. The pathophysiology of HPS remains unclear; however, a “cytokine storm” has been assumed as a cause in one theory (21, 22). Currently, there is no specific diagnostic method for HPS, although the diagnostic standard revised by the Histiocyte Society in 2004 has been widely adopted (4). The diagnosis can be made according to any of the following.

Molecular diagnosis: At present, it is known that related genes, such as *PRF1, UNC13D, STX11, STXBP2, Rab27a, LYST*, and *SH2D1A*, have pathological mutations; and meet five of the following eight standards: fever, temperature > 38.5°C, duration > 7 days; spleen, hypocytopenia (two or three series of peripheral blood); hemoglobin < 90 g/L, PLT < 100 × 10^9^/L, and GR < 1.0 × 10^9^/L, and these are not caused by the decreased hematopoietic function of bone marrow; hypertriglyceridemia and/or hypoproteinemia: triacylglycerol > 3 mmol/L, fibrinogen < 1.5 g/L; phagocytic blood cells found in bone marrow, spleen, or lymph nodes; elevated serum ferritin ≥ 500 μg/L; decreased level or lack of NK cell activity; and an increase in the level of sCD25.

Although the general prognosis for HPS is poor, the prognosis for HPS caused by bacterial infection is better. However, the prognosis for HPS caused by Epstein–Barr virus is the worst. The mortality of HPS caused by other viruses is generally ~50%, and the mortality of tumor-related HPS is almost 100%. The appearance of HPS indicates the deterioration of this disease. The main causes of death include hemorrhage, infection, multiple organ failure, and disseminated intravascular coagulation. Factors associated with poor prognosis include low albumin and/or high lactate dehydrogenase levels, severe hemorrhage and uncontrolled infection, thrombocytopenia, liver function damage, and aging (23). HPS therapies can be divided into two categories: induced remission and etiological therapies. Induced remission therapy mainly refers to controlling excessive inflammation, while the objective of etiological therapy is to correct immunodeficiency by actively treating the primary disease (24). In this case, after the treatment with etoposide and hormone, the temperature of the patient rapidly returned to normal ranges, and the related indices were improved. In summary, the clinical manifestations of HPS are complex and diverse, and the disease progresses rapidly and the prognosis is poor. Therefore, it is significant to make early diagnosis for this disease and treat it correctly.

Although HPS has been investigated in some studies around the world, cases of *C. psittaci* pneumonia concomitant with secondary HPS during pregnancy have not, to our knowledge, been reported. Katsura et al. reported a case of psittacosis during pregnancy, and reviewed the research about 23 cases of psittacosis during pregnancy from 1967 to date (25). The mortality rates for fetuses and pregnant women were 82.6% (19/23) and 8.7% (2/23), respectively. The primary reason for the death of pregnant women and fetuses was that *C. psittaci* infection was not diagnosed in time during the progression of the disease. In this case, although the fetus was subjected to labor induction, the reason for the better prognosis of the patient was that the diagnosis of *C. psittaci* pneumonia was confirmed by mNGS in the early stage of the disease, which provided a basis for the selection of effective antibiotics to quickly control lung lesions. In those patients with a persistent high fever after pulmonary lesions are obviously mitigated, HPS is diagnosed by bone marrow biopsy, smear, NK cell activity, soluble CD25 level, and a CD107a challenge test. The condition of the patient was improved after the active treatment with etoposide and hormone therapy according to the HLH-94 and HLH-2004 guidelines, and all indicators gradually returned to normal ranges.

Through this study, it can be recognized that the clinical application of mNGS should be actively pursued for pulmonary infections with unknown etiology.

## Data Availability Statement

The original contributions presented in the study are included in the article, further inquiries can be directed to the corresponding author.

## Ethics Statement

The studies involving human participants were reviewed and approved by the Ethics Review Board at First affiliated hospital of Anhui Medical University. The patients provided their written informed consent to participate in this study. Written informed consent was obtained from the individual for the publication of any potentially identifiable images or data included in this article.

## Author Contributions

RW and LS conception and design of the study. LS, BP, and PW provided clinical treatment for the patient and manuscript preparation. RW performed manuscript review. All authors have read and approved the content of the manuscript.

## Funding

The present study was supported by a fund from the Natural Science Foundation of China (No. 81970051). Excellent Top Talent Cultivation Project of Anhui Higher Education Institutions (gxgwfx2021014).

## Conflict of Interest

The authors declare that the research was conducted in the absence of any commercial or financial relationships that could be construed as a potential conflict of interest.

## Publisher's Note

All claims expressed in this article are solely those of the authors and do not necessarily represent those of their affiliated organizations, or those of the publisher, the editors and the reviewers. Any product that may be evaluated in this article, or claim that may be made by its manufacturer, is not guaranteed or endorsed by the publisher.
